# The Circadian Rhythm of Copeptin, the C-Terminal Portion of Arginine Vasopressin

**DOI:** 10.1155/2017/4737082

**Published:** 2017-06-01

**Authors:** Svetlana Beglinger, Jürgen Drewe, Mirjam Christ-Crain

**Affiliations:** ^1^Department of Endocrinology, Diabetes and Metabolism, University Hospital of Basel, 4031 Basel, Switzerland; ^2^Department of Clinical Pharmacology, University Hospital of Basel, 4031 Basel, Switzerland

## Abstract

**Background:**

Several studies have investigated copeptin as a prognostic marker of different acute diseases and as a diagnostic marker in disorders of water and salt homeostasis. However, no data of the normal circadian rhythm of copeptin in healthy subjects are available.

**Aim:**

To investigate the circadian rhythm of copeptin in healthy subjects under standardized conditions.

**Methods:**

19 healthy volunteers aged 18 to 53 years, male and female, were studied in a prospective observational study. In all 19 participants, blood samples for copeptin were taken in regular intervals of 30 minutes for 24 hours after a fasting period of minimum 8 hours.

**Results:**

The mean values of copeptin showed a circadian rhythm, similar to that described for AVP release, with a trend towards higher levels (5.9 ± 1 pmol/L) at night and early morning between 4 am and 6 am and lowest levels (2.3 ± 0.2 pmol/L) in the late afternoon between 5 pm and 7 pm. This finding was only observed in individuals with initial higher copeptin levels, whereas in individuals with lower basal copeptin levels no circadian rhythm was observed.

**Conclusion:**

There is evidence for a circadian rhythm in copeptin release during 24 hours, however, of minor extent. These findings suggest that copeptin levels can be determined irrespectively of the time of the day.

## 1. Introduction

Copeptin, a 39-amino acid glycopeptide comprising the C-terminal part of the AVP precursor (CT-proAVP (Arginine Vasopressin)), was found to be a stable and sensitive marker for AVP release and seems to be released stoichiometrically together with AVP [1]. Copeptin shows similar changes during disordered water states or osmolality as previously shown for AVP [2, 3]. AVP is a key hormone in water homeostasis. It is synthesized in the hypothalamus and secreted by the neurohypophysis into the blood [4]. The main stimulus for AVP secretion is hyperosmolality and decrease in blood volume [4]. Alternative stimuli are severe stress in the context of myocardial infarction, stroke, brain injury, and sepsis [5–8]. In contrast to AVP, which is technically difficult to measure because of its instability, copeptin is stable for days at room temperature and can be detected within 3 hours [9]. 

Consequently, copeptin is commonly used as a prognostic marker in myocardial infarction and hemorrhagic and septic shock in adults [10] and as a diagnostic marker in disorders of water and salt homeostasis [9]. However, only little is known about physiological fluctuations of copeptin levels and about its circadian rhythm. So far, only preliminary data in 7 individuals suggest no consistent circadian rhythm of copeptin [11]. For AVP a subtle circadian release pattern has been observed with highest levels at night between 24.00 h and 02.00 h and lowest levels in the afternoon at 16.00 till 20.00 h [12].

Therefore, the aim of our study was to investigate the circadian release pattern of copeptin under standardized conditions.

## 2. Subjects and Methods

### 2.1. Subjects

19 male and female subjects, aged 18–53 years (mean 25.8 years), participated in the study. Body weight of all subjects was within the normal range for age, sex, and height, and the BMI was in normal range between 18.5 and 24.9 kg/m^2^. Each subject gave written informed consent for the study. The human ethics committee of the University Hospital Basel, Switzerland, approved the protocol. Before acceptance, each participant was required to complete a medical interview, received a full physical examination, and participated in an initial laboratory screening. In female participants the hemoglobin blood count had to be 125 g/L or more and in male participants 130 g/L or more, according to the standing orders of the blood donation center of the University Hospital Basel. Subjects receiving any medication (especially birth control pill in female) or with a history of chronic illness (especially heart insufficiency, renal insufficiency, and diabetes) or chronic psychiatric disease or pregnant women were excluded.

### 2.2. Methods

The participants were asked not to do excessive sports and not to drink more than 2000 mL three days before the examination day and to keep a diary about their drinking volume. All participants arrived to the ward after an overnight fast of minimum 8 hours, where they received an assessment of clinical parameters and an intravenous line in the antecubital fossa of the arm. Regular blood samples were taken every 30 minutes within 24 hours. At the same time points, clinical parameters were assessed.

During the blood sampling the participants received standardized meals of the hospital for breakfast, lunch, and dinner. The participants were allowed to drink 1800 mL/day, but not more than 2 dL at one time and not more than 3 dL per hour. An intravenous infusion with isotonic solution NaCl 0.9% was inserted only when the intravenous line was difficult to flush with a basal rate of 10 mL/h with a maximum of 240 mL/24 h. During the 24 hours of blood sampling the participants were not allowed to walk around or do stronger physical examination. Visiting the toilet was allowed as well as sitting in the bed during mealtime. All participants underwent normal daily routine with light activities and normal exposure to daylight through windows and darkness during nighttime including dimout with curtains and roller shutters.

After completing the 24 hours of blood sampling the participants received another measurement of the hemoglobin blood count. After receiving a breakfast the study participation was completed.

Cortisol was measured concomitantly with copeptin because cortisol is known to show a circadian rhythm with high levels in the morning and low levels at night. Therefore, we used cortisol levels for validation of the circadian rhythm under standardized conditions.

### 2.3. Biochemistry

Copeptin was collected in a 7.5 mL Monovette® EDTA KE and AVP in a 2.7 mL Monovette Heparin. Every 3 hours a tube of 4.7 mL heparin plasma for sodium and osmolality was collected. Copeptin was measured with an automated immunofluorescent assay for the quantitative determination of copeptin (Kryptor Compact Plus, Thermo Scientific B.R.A.H.M.S, Hennigsdorf, Germany) with a lower detection limit of 0.69 pmol/L.

Sodium and osmolality were quantified immediately after blood withdrawal with a “ion selective electrode” ISE (Roche Diagnostics) by the Central Laboratory of the University Hospital Basel.

Cortisol was measured with ElektroChemiLumineszenz ImmunoAssay “ECLIA” Elecsys and cobas e Immunoassay System with a lower detection limit of 0.500 nmol/L.

### 2.4. Statistics

Maximum (*C*_max_) and minimum (*C*_min_) copeptin and the time points of their occurrence, *T*_max_ and *T*_min_, respectively, were determined by inspection of the data. The area under the copeptin concentration time curve over the 24 h observation period (AUC (0–24)) was calculated by the linear trapezoidal rule. The average copeptin concentration was estimated as follows: *C*_average_ = AUC/duration of observation period. Fluctuation around *C*_average_ was calculated as the fluctuation index (FI): FI = (*C*_max_ − *C*_min_)/*C*_average_ × 100 (%).

Correlation of individual parameters was done by linear regression analysis (Origin software, version 9.0, OriginLab, Northampton, MA, USA). Statistical comparisons within and between subjects were done with the two-sided paired and unpaired *t*-tests, respectively (SPSS for Windows, version 24).

## 3. Results

### 3.1. Baseline Characteristics

19 healthy nonsmoking volunteers (10 female, 9 male) were included in the study.

The mean age was 25.8 years (18–53 years), mean weight 70.7 ± 2.2 kg, and mean BMI 22.7 ± 0.1 kg/m^2^. Physical examination and medical history were normal; 4 women were on regularly birth control pills. There were no differences seen in the copeptin concentrations between males and females.

### 3.2. Circadian Rhythm of Cortisol

Cortisol levels were significantly (*p* < 0.001, paired *t*-test) higher in the morning (8 am) than at night (12 pm), that is, 415.8 ± 62.3 nmol/L and 78.9 ± 3.3 nmol/L, respectively.

### 3.3. Circadian Rhythm of Copeptin

**Figure 1:**
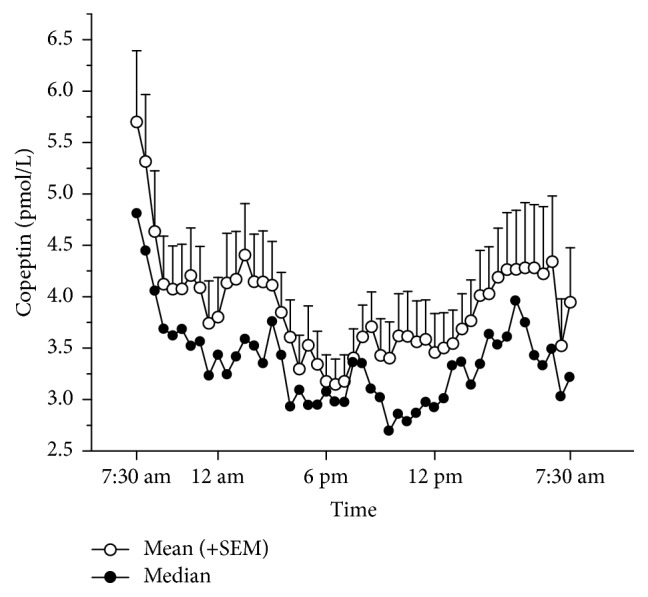
*Open circles:* mean (+SEM) and* filled circles:* median plasma concentrations of copeptin (pmol/L) of all subjects over a 24-hour period.

The mean and median copeptin levels are shown in Figure 1.

Baseline copeptin levels at 7.30 am ranged within 2.56–12.6 pmol/L (mean 5.7 ± 0.7 pmol/L). Copeptin *C*_max_ levels were the highest in the morning, with the maximum value of 5.9 ± 1 pmol/L at 4.42 am, and were the lowest between 5.18 pm and 7 pm with the lowest level (*C*_min_) of 2.3 ± 0.2 pmol/L.

**Figure 2:**
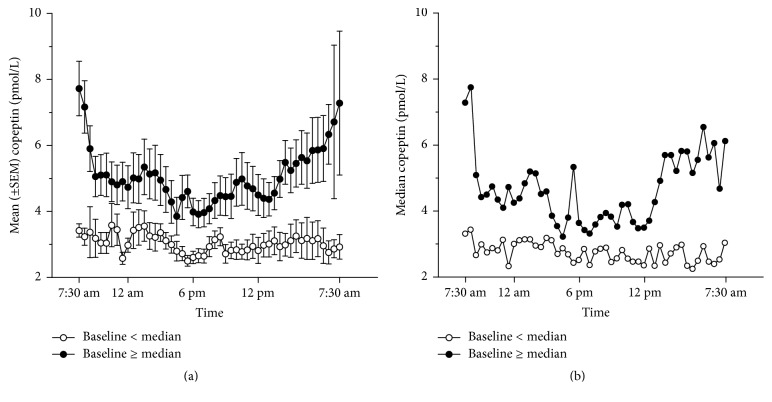
Mean (±SEM) (a) and median (b) plasma concentrations of copeptin (pmol/L) over a 24-hour period. Full circles: patients with baseline copeptin level ≥ median baseline level (e.g., 4.8 pmol/L). Open circles: patients with baseline copeptin level < median (e.g., 4.8 pmol/L).

**Figure 3:**
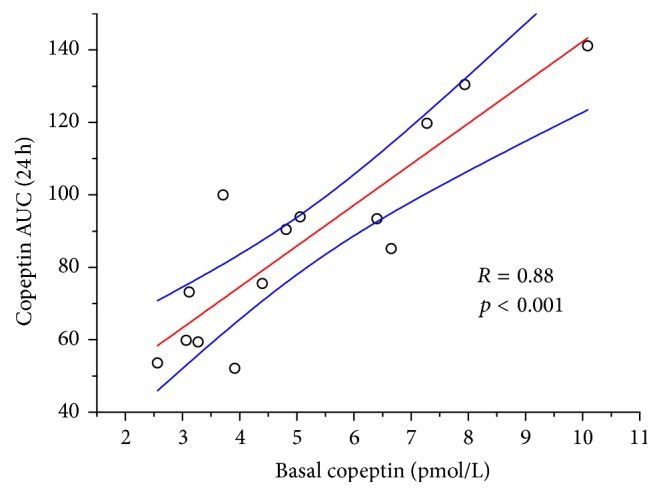
Linear regression analysis of baseline copeptin concentrations versus copeptin AUC (0–24 h). *R* = coefficient of regression, *p* = level of significance, red line regression line, blue lines = 95% confidence bands of the regression line.

Therefore the mean values of copeptin showed a certain circadian rhythm with a trend towards higher levels at night and early morning between 4.42 and 5.54 am and lowest levels in the late afternoon (Figure 1). This finding was primarily seen in those individuals with high initial basal copeptin levels (e.g., ≥ median baseline value of 4.8 pmol/L; subpopulation A) (Figures 2(a) and 2(b)), whereas in individuals with a basal copeptin below the median level (subpopulation B) no circadian rhythm was observed; that is, baseline copeptin levels were predictive for the release pattern (see Figure 3). These two subpopulations differed significantly in many pharmacokinetic parameters (see Table 1): Compared to patients from subpopulation B, patients from subpopulation A had higher AUC values (*p* = 0.003), higher morning *C*_max_ values (*p* = 0.043), higher *C*_min_ values (*p* = 0.014), and higher *C*_average_ values (*p* = 0.003). The more expressed circadian rhythm of patients from subpopulation A was reflected by significantly increased fluctuation index (*p* = 0.033). Although partly significantly different between the subgroups, *T*_max_ and *T*_min_ values were of limited relevance since the location of *C*_max_ and *C*_min_ was almost at random for subjects displaying no circadian rhythm, and mean values were prone to bias induced by outliers.

There were no statistically significant differences between males and females in the parameters of copeptin.

Individuals with higher baseline copeptin levels showed significantly higher AUC (24 h) values (*R* = 0.88, *p* < 0.001; Figure 3).

In contrast, there was no significant correlation of copeptin values with sodium (*R* = 0.04, *p* = 0.62), osmolality (*R* = −0.05, *p* = 0.55), BMI (*R* = 0.23, *p* = 0.67), and age (*R* = −0.29, *p* = 0.99) during the 24 hours of testing.

## 4. Discussion

The main finding of our study is that copeptin shows a circadian rhythm similar to that described for AVP, but with a later peak for *C*_max_ and later nadir for *C*_min_. This finding might be due to the longer half-life of copeptin of around 86 minutes compared to the shorter half-life of AVP with 44 minutes or less (13; own data submitted). The observed rhythm, however, is of only minor extent and counts only for individuals with high basal copeptin levels.

The measurement of cortisol shows, as expected, a strong circadian rhythm with a nadir at midnight and increasing levels at 8 am [13–15], validating our study protocol for circadian rhythmicity.

AVP has been shown to have a minor circadian rhythm with maximum levels between midnight and 2 am [12]. In our study copeptin levels showed a maximum at 4.42 am and a minimum at about 6 pm in the group with a circadian rhythm (subpopulation A).

The reason why only individuals with high median copeptin levels show a circadian rhythm is unclear. We can only speculate that different chronotype profiles in humans with either morning or evening or neither chronotype, might impact the diurnal release pattern of copeptin. It was found that morning chronotype subjects had higher cortisol levels after awakening than evening or neither types with more pronounced diurnal rhythm [16]. Although we observed a certain rhythm in some of the individuals, the minor difference of minimum and maximum copeptin levels suggests that copeptin levels can be determined irrespectively of time of the day. This stands in contrast to cortisol levels where specific times of day are needed for diagnostic purposes.

Our study has strengths and limitations. A strength of our study is the relative high number of volunteers and the standardized study protocol with a diary, where the individuals noticed their exercise and fluid intake during three days prior to the start of the examination and the standardized fluid and meal intake during the study.

A limitation is that our design implies an artificial study setting. However, in our opinion this is the only possible study protocol to evaluate the circadian rhythm of copeptin. Second, we did not measure AVP levels, which prohibits a direct comparison between copeptin and AVP levels. Third, copeptin values are increased in stress situations; therefore the participants had 15 minutes of rest before installing the intravenous line. Nevertheless, we cannot exclude entirely that the first copeptin levels have not been influenced by stress. Fourth, we are aware that there is an imprecision profile in the low molecular range, although our chosen method has a low detection limit. Fifth, we are aware that plasma melatonin is known as even more valuable parameter of choice for evaluation of individual chronobiology. Nevertheless, we used plasma cortisol for proof-of-concept of the circadian rhythm, because of its use in daily routine and better stability at room temperature and lower costs.

## 5. Conclusion

This study shows for the first time the release pattern of copeptin during 24 hours in healthy subjects under standardized conditions. Thereby, copeptin underlies a circadian rhythm of minor extent and in only some individuals. This suggests that there is no defined dependency on time of the day for its measurement in clinical routine, which strengthens the importance of copeptin as biomarker for several diseases.

## Figures and Tables

**Table 1:** Kinetic parameters of copeptin.

	AUC (24 h) pmol × h/L	*C*_min_^(1)^ pmol/L	*T*_min_^(1)^ h	*C*_max_^(1)^ pmol/L	*T*_max_^(1)^ h	*C*_average_ pmol/L	Fluctuation index %	Baseline copeptin pmol/L
*All subjects*								
Mean ± SEM	87.7 ± 7.5	2.3 ± 0.2	5.18 pm ± 1.1	5.9 ± 1.0	4.42 am ± 0.6 h	3.7 ± 0.3	120.5 ± 13.6	5.7 ± 0.7
Median (range)	87.8 (52.2–141.1)	2.2 (1.5–5.2)	6.30 pm (5.30–12.00 pm)	4.5 (2.4–19.4)	5.00 am (0.00–7:30 am)	3.7 (2.2–5.9)	113 (72.6–328.5)	4.8 (2.6–12.6)

*Subpopulation A: baseline values ≥ median*								
Mean ± SEM	107.7 ± 8.4	2.8 ± 0.4	7.00 pm ± 0.7	8.7 ± 1.7	5.54 am ± 0.9	4.6 ± 0.4	163.4 ± 33.4	7.7 ± 0.8
Median (range)	93.0 (85.2–141.1)	2.5 (1.6–52)	7:00 pm (4:00–10.5 pm)	7.4 (3.3–19.4)	6.48 am (0.00–7.30 am)	3.9 (3.5–5.9)	144.9 (113.8–328.5)	7.3 (4.81–12.6)

*Subpopulation B: baseline values < median*								
Mean ± SEM	67.7 ± 6.4	1.9 ± 0.1	3.48 pm ± 1.8	3.7 ± 0.4	3.30 am ± 0.9	2.8 ± 0.3	92.9 ± 6.1	3.4 ± 0.2
Median (range)	59.9 (52.2–100.0)	1.8 (1.5–3.0)	4.00 pm (8.30 am–12.00pm)	3.2 (2.4–6.5)	3.00 am (0.00–7:30 am)	2.5 (2.2–4.2)	88.9 (72.6–113.4)	3.3 (2.6–4.4)

*p*-value^(2)^	0.003	0.014	NS	0.043	NS	0.003	0.033	0.033

SEM = standard error of the mean; ^(1)^measured for the period from 7.5 am to 7.5 am (24 hours); ^(2)^comparing the subgroups (e.g., baseline ≥ median versus baseline < median).

## Abbreviations

AVP: Arginine vasopressin
AUC: Area under curve
BMI: Body mass index.
